# Differential regulation of MAGE-A1 promoter activity by BORIS and Sp1, both interacting with the TATA binding protein

**DOI:** 10.1186/1471-2407-14-796

**Published:** 2014-11-03

**Authors:** Heidi Schwarzenbach, Corinna Eichelser, Bettina Steinbach, Josefine Tadewaldt, Klaus Pantel, Victor Lobanenkov, Dmitri Loukinov

**Affiliations:** Department of Tumor Biology, University Medical Center Hamburg-Eppendorf, Martinistraße 52, Hamburg, 20246 Germany; Laboratory of Immunogenetics, National Institute of Allergy and Infectious Disease, National Institutes of Health, Rockville, Maryland USA

**Keywords:** DNA methylation, Histone modifications, Promoter activation, Protein protein interaction

## Abstract

**Background:**

As cancer-testis MAGE-A antigens are targets for tumor immunotherapy, it is important to study the regulation of their expression in cancers. This regulation appears to be rather complex and at the moment controversial. Although it is generally accepted that MAGE-A expression is controlled by epigenetics, the exact mechanisms of that control remain poorly understood.

**Methods:**

We analyzed the interplay of another cancer-testis gene, BORIS, and the transcription factors Ets-1 and Sp1 in the regulation of MAGE-A1 gene expression performing luciferase assays, quantitative real-time PCR, sodium bisulfite sequencing, chromatin immunoprecipitation assays and pull down experiments.

**Results:**

We detected that ectopically expressed BORIS could activate and demethylate both endogenous and methylated reporter MAGE-A1 promoter in MCF-7 and micrometastatic BCM1 cancer cell lines. Overexpression of Ets-1 could not further upregulate the promoter activity mediated by BORIS. Surprisingly, in co-transfection experiments we observed that Sp1 partly repressed the BORIS-mediated stimulation, while addition of Ets-1 expression plasmid abrogated the Sp1 mediated repression of MAGE-A1 promoter. Both BORIS and Sp1 interacted with the TATA binding protein (hTBP) suggesting the possibility of a competitive mechanism of action between BORIS and Sp1.

**Conclusions:**

Our findings show that BORIS and Sp1 have opposite effects on the regulation of MAGE-A1 gene expression. This differential regulation may be explained by direct protein-protein interaction of both factors or by interaction of MAGE-A1 promoter with BORIS alternatively spliced isoforms with different sequence specificity. We also show here that ectopic expression of BORIS can activate transcription from its own locus, inducing all its splice variants.

**Electronic supplementary material:**

The online version of this article (doi:10.1186/1471-2407-14-796) contains supplementary material, which is available to authorized users.

## Background

Based on their pronounced tumor specificity, cancer-testis antigens (CTA) which comprise numerous gene families, such as MAGE-A, are particularly promising targets for specific anti-cancer immunotherapy. Clinical studies have demonstrated vaccination-induced T-cell mediated responses in cancer patients by CTA [1]. The MAGE-A gene family comprising 12 members (MAGE-A1-12) is located on chromosome X [2]. With the exception of testicular germ cells (spermatogonia and primary spermatocytes) and placenta, they are silent in normal somatic tissues, but expressed in numerous epithelial carcinomas and leukemia [3]. Nevertheless, the MAGE-A protein levels can vary widely in tumors, and not all tumors express these antigens. Previous studies revealed that control of MAGE-A expression is rather complex and to a large extent poorly understood. The restricted expression pattern of MAGE-A antigens is regulated by epigenetic mechanisms [4]. Methylation of CpG dinucleotides on the MAGE-A1 promoter prevents access of transcription factors Ets-1 and Sp1 to their binding sites which are responsible for the transcriptional activation of MAGE-A genes [5]. Histone deacetylation, leading to a compact and transcriptionally inactive chromatin structure, also contributes to the repression of MAGE-A genes [6].

In general, histones are subject to post-translational modifications, such as acetylation, phosphorylation, ubiquitination and methylation [7]. Deacetylation of acetylated N-terminal tails of histones in active chromatin regions occurs through histone deacetylases (HDACs) [8]. Methylation of the lysine residue 4 of histone H3 (H3K4) is highly conserved and associated with transcriptionally active genes. Methylation of the lysine residue 9 of histone H3 (H3K9) recruits the heterochromatin protein HP-1, which condenses chromatin into an inactive conformation [9]. Both DNA methylation and histone modifications may be linked by methyl-CpG binding proteins (MBDs). Nearly all members of the MBD family can interact with histone methyltransferases and deacetylases. To date, five MBDs (MBD1, MBD2, MBD3, MBD4 and MeCP2) have been identified and are involved in the transcriptional repression of methylated DNA [10]. We observed that among the MBDs, the variant MBD1v1 of the five MBD1 isoforms has the ability to repress the unmethylated MAGE-A1 promoter and downregulate Ets-mediated transcriptional activation [11]. This MBD1v1-mediated downregulation of MAGE-A1 gene expression is dependent on three CXXC domains, which additionally repress unmethylated promoters [12]. Conversely, we showed that MBD2a may enhance the basal promoter activity of MAGE-A1 [11]. In line with our observation, a previous report demonstrated that the longer form of MBD2, the isoform MBD2a, is not only involved in gene repression but also in promoting activation of the unmethylated cAMP-responsive genes by interaction with the RNA helicase A, and accordingly, MBD2a may be either a transcriptional activator or repressor [13].

The ectopic expression of BORIS (Brother of the Regulator of Imprinted Sites), the mammalian CTCF paralog, may induce the expression of MAGE-A1 gene [14]. Like MAGE-A1, BORIS is a CTA, and in addition to its normal expression in male germ cells, BORIS is expressed in various solid tumors, with frequent co-expression of other CTAs [15]. The transcription of BORIS is regulated by three alternative promoters (A, B, C) utilizing five distinct 5´UTRs (untranslational regions) [16]. So far, 23 BORIS splice variants with distinct expression profiles in normal germ line and cancer cells have been characterized, exhibiting differential DNA binding activities and varying transcriptional properties. These alternative transcripts have the potential to encode 17 distinct proteins with varying number of zinc fingers in the DNA binding domain and different combinations of amino- and carboxy-termini. *In vitro* binding of BORIS isoforms to DNA targets can be methylation-sensitive and depends on the number and specific composition of zinc fingers. Nine of the 17 *in vitro* translated BORIS isoproteins bound the H19 ICR CTCF target site, whereas the remaining other 8 BORIS isoforms did not. The presence of a specific long amino terminus in the different isoforms is necessary and sufficient to activate the testis-specific cerebroside sulfotransferase (CST) transcription. Accordingly, isoforms B2, B3, B4 and B5 lacking this long amino terminus could bind to CST, but did not induce transcription above background level [17]. Recent experiments in cell lines suggested that BORIS expression is sufficient to simultaneously demethylate and activate the transcription of CTAs and oncogenes [14, 18, 19]. However, analyses of melanoma tissue samples, where MAGE-A1 may be expressed in the absence of BORIS, indicated that MAGE-A1 expression can also be induced by other mechanisms [20]. In addition to its role as a putative component in aberrant DNA demethylation and transcriptional activation, BORIS may also participate in histone demethylation and chromatin remodelling [21, 22].

In the current study, we investigated the role of BORIS in the context of transcription factors Ets-1 and Sp1, known to be implicated in MAGE gene regulation, in the activation of MAGE-A1 expression. We found that BORIS can activate MAGE-A1, both at the endogenous transcript level and in reporter assays. Ectopic Sp1 expression partly abrogates this BORIS-induced activation, while ectopic Ets-1 lifts the repressive effect of Sp1. Interaction of both BORIS and Sp1 with the TATA binding protein (hTBP) is also established in our manuscript. Moreover, the impact of BORIS on the epigenetic signature associated with the MAGE-A1 promoter and its interaction with the transcription factors were analyzed.

## Methods

### Cell lines and drug treatment regimens

The cancer cell lines MDA-MB-468 and MCF-7 (breast adenocarcinoma) were cultured in DMEM (Invitrogen, Karlsruhe, Germany) supplemented with 10% FCS (fetal calf serum; PAA Laboratories, Cölbe, Germany) and 2 mM L-glutamin (Invitrogen) under standard conditions (37°C, 10% CO_2_, humidified atmosphere). The micrometastatic BCM1 (breast cancer) cells [23, 24] were cultured at 37°C, 5% CO_2_ and 10% O_2_ in RPMI (Invitrogen, Karlsruhe, Germany) supplemented with 10% FCS (PAA Laboratories), 2 mM L-glutamin (Invitrogen), 10 mg/mL Insulin-Transferrin-Selenium-A (Invitrogen), 50 ng/mL recombinant human epidermal growth factor, and 10 ng/mL human basic fibroblast growth factor (Miltenyl Biotec, Bergisch-Gladbach, Germany). Cell viability was determined by trypan blue staining. MCF-7 and BCM1 cells were stimulated by 5-aza-2´-deoxycitidine (5-aza-CdR, f.c. 1 μM, Sigma-Aldrich, Steinheim, Germany) for 72 h. 5-aza-CdR-treated or untreated cells were stimulated by Trichostatin A (TSA, f.c. 500 nM, Sigma-Aldrich) for 24 h after 48 hour incubation with or without 5-aza-CdR.

### RT-PCR

For cloning of the transcription factors Ets-1, Sp1, and hTBP, total RNA was prepared using the RNeasy® Mini Kit (Qiagen, Hilden, Germany) and performed according to the manufacturer’s description. Synthesis of cDNA was carried out using the First-strand cDNA synthesis kit and priming with the oligonucleotides dT (Fermentas, St. Leon-Rot, Germany). PCR amplification of cDNA was performed with primers specific for Ets-1: 5´-CCA AAA TGG TAC CAT GAA GGC GGC CGT CGA T-3´ and 5´-GAA TCA AGC GGC CGC TCA CTC GTC GGC ATC TGG-3´; Sp1: 5´- CCA AAA TGA ATT CAT GAG CGA CCA AGA TCA C-3´ and 5´-GAA TCA ACT CGA GTC AGA AGC CAT TGC CAC T-3´; full length, N-terminal and C-terminal hTBP: 5´-CCA AAA TGA ATT CAT GGA TCA GAA CAA CAG C-3´, 5´-GAA TCA ACT CGA GTT ACG TCG TCT TCC TGA ATC C-3´, 5´-GAA TCA ACT CGA GAG AAC TCT CCG AAG CTG G-3´ and 5´-CCA AAA TGA ATT CGG GAT TGT ACC GCA GCT G-3´; BORIS: 5´-CTCAGGTGAGAAGCCTTACG-3´ and 5´-TGA TGG TGG CAC AAT GGG-3´. The reaction was in a final volume of 20 μl containing PCR buffer (Qiagen), 200 μM of each dNTP (Roche Applied Science, Mannheim, Germany), 0.5 μM of each primer and 2.5 units of Pfu turbo hot start polymerase (Stratagene, Amsterdam, Netherlands). Template DNA was amplified in 35 cycles. The PCR products were separated on a 1% agarose gel.

### Vector constructions

For transient transfections the MAGE-A1 promoter region fragment (-77/+183) containing the BORIS binding site downstream of the transcriptional start site was amplified in a PCR using the following primer pair: 5’-GTT CCC GCC AGG AAA CAT C-3’ and 5’-GCC CAG GCT GAG ACG TCT TCC-3’. After amplification the PCR product was cloned into a pCR2.1 TOPO vector (Invitrogen), digested with the restriction enzymes KpnI and XhoI and subcloned into the corresponding restriction sites of the pGL2-Luciferase reporter plasmid (Promega). For the construction of the expression plasmids, we cloned cDNA of Ets-1 into KpnI and NotI, and of Sp1, full length, N-terminal and C-terminal hTBP into EcoRI and XhoI sites of the pcDNA3.1 vector (Invitrogen). The pBIG-HA BORIS plasmid containing the full-length BORIS sequence was described in [14].

To analyze protein-protein interactions, we amplified the sequences of Ets-1, Sp1, MBD1v1, MBD2b, hTBP-full length, hTBP-N and hTBP-C of pcDNA3.1 expression constructs and BORIS of pBIG-HA construct by primers containing the restriction sites SgfI and PmeI. Following specific primers were used for Ets-1: 5’-CCA AAA TGC GAT CGC ATG AAG GCG GCC GTC GAT-3’ and 5’-GAA TCA AGT TTA AAC TCA CTC GTC GGC ATC TGG-3’, Sp1: 5’-CCA AAA TGC GAT CGC ATG AGC GAC CAA GAT CAC-3’ and 5’-GAA TCA AGT TTA AAC TCA GAA GCC ATT GCC ACT-3’, MBD1v1: 5’-CCA AAA TGC GAT CGC ATG GCT GAG GAC TGG CT-3’ and 5’-GAA TCA AGT TTA AAC CTA CTG CTT TCT AGC TC-3’, MBD2b: 5’-CCA AAA TGC GAT CGC ATG GAT TGC CCG GCC CTC-3’ and 5’-GAA TCA AGT TTA AAC TTA GGC TTC ATC TCC ACT-3’, full length hTBP: 5’-CCA AAA TGC GAT CGC ATG GAT CAG AAC AAC AGC-3’ and 5’-GAA TCA AGT TTA AAC TTAC GTC GTC TTC CTG AA-3’, N-terminal hTBP: 5’-CCA AAA TGC GAT CGC ATG GAT CAG AAC AAC AGC-3’ and 5’-GAA TCA AGT TTA AAC AGA ACT CTC CGA AGC TGG-3’, C-terminal hTBP: 5’-CCA AAA TGC GAT CGC GGG ATT GTA CCG CAG CTG-3’ and 5’-GAA TCA AGT TTA AAC TTAC GTC GTC TTC CTG AA-3’, and BORIS: 5’-CCA AAA TGC GAT CGC ATG TAC CCA TAC GAT GTT CCA-3’ and 5’-GAA TCA AGT TTA AAC TCA CTT ATC CAT CGT GTT-3’. After PCR and gel purification, the fragments were inserted into pCR2.1 TOPO-vector (Invitrogen), cleaved by the restriction enzymes SgfI and PmeI, and cloned into pFN19A (HaloTag®7) T7 SP6 Flexi vector (Promega). All clones were verified by restriction digestion and DNA sequencing.

### *In vitro*methylation of plasmid DNA

Twenty μg of reporter plasmids containing the MAGE-A1 promoter fragment were methylated by HpaII methylase (New England Biolabs, Schwalbach, Germany) for 4 h at 37°C in the presence of the co-factor SAM (S-Adenosyl methionine, New England Biolabs). The methylation efficiency of plasmid DNA was confirmed by restriction enzyme digestion with HpaII (New England Biolabs). A control digest was done using the isoschizomer MspI (New England Biolabs).

### Transient transfection and luciferase assay

MDA-MB-468, MCF-7 and BCM1 cells were transiently transfected with 0.5 μg of reporter plasmids (unmethylated or HpaII-methylated) and pcDNA3.1 expression plasmids up to 2 μg using FuGENE HD Reagent (Roche Applied Science, Mannheim, Germany) in a 6-well plate (BD Falcon, Heidelberg, Germany). For efficiency control 0.2 μg of a vector encoding for Renilla Luciferase (Promega, Mannheim, Germany) was co-transfected. Cells were cultured for 48 h under standard conditions. Luciferase assays were performed using the Dual-Luciferase Reporter Assay System kit (Promega) according to the manufacturer’s protocol. Promoter-driven luciferase activity was measured on a 20/20^n^ Luminometer Turner Biosystems (Promega) and normalized by the Renilla luciferase activity. Each transfection experiment was carried out in duplicate wells and repeated several times.

### Transient transfection and mRNA expression analyses

To determine the mRNA expression of MAGE-A1 in MDA-MB-468, MCF-7 and BCM1 cells, transient transfections were performed using 5 μg expression plasmids and FuGENE HD Reagent (Roche Applied Science). After a 72 hour transfection total RNA was isolated using the RNeasy® Mini Kit (Qiagen) according to the manufacturer’s protocol. RNA was converted into cDNA using the First Strand cDNA Synthesis kit and oligo(dt) primers (Fermentas). Two μL of cDNA (2 μg) were amplified in a 20-μl final volume containing PCR buffer (Qiagen), 200 μM of each dNTP (Roche Applied Science), 0.5 μM of each primer and 2.5 units of Taq DNA polymerase (Qiagen). The MAGE-A primer pairs for PCR have been previously described [6]. The reaction was run for 35 cycles on a Thermal Cycler (Flexigene, Techne, Stafordshire), and the PCR products were electrophoretically separated on a 1% agarose gel.

To degrade the BORIS mRNA and consequently, inhibit its protein expression, 1 μg expression plasmid containing the BORIS sequence and/or 1 μg plasmid containing the BORIS specific shRNA cassette and/or 1 μg control plasmid encoding for a scramble shRNA were transfected in BCM1 cells using FuGENE HD Reagent (Roche Applied Science) in a 6-well plate (BD Falcon). After a 48 or 72 h transfection total RNA was extracted and converted into cDNA. Two μl of cDNA (0.5 μg) were amplified in a quantitative real-time PCR.

### FACS (Fluorescence Activated Cell Sorting) analyses

2×10^7^ MCF-7 cells transfected with 5 μg pBIG-HA Boris expression plasmid and 10 μl XtremeGene HP (Roche Applied Science) were washed in 10 ml staining buffer (0.1% BSA, 0.1% sodium azide in PBS). Following incubation of the transfected and non-transfected cells with 50 μl FcR blocking reagent (Miltenyl Biotec) for 15 min at 4°C and washing in 10 ml staining buffer, the cells were fixed in 500 μl IC Fixation buffer (eBioscience, Frankfurt, Germany) in the dark for 20 min. The cells were washed twice in permeabilization buffer (eBioscience) and incubated with 4 μg of anti-Boris primary antibody or purified mouse IgGk isotype control antibody (BD Biosciences, Heidelberg, Germany) for 30 min at 4°C. After washing, the cells were incubated with 4 μg of FITC conjugated IgG/IgM goat anti-mouse secondary antibody (BD Biosciences) in the dark for 30 min at 4°C. The washed cells were filtered through a 30-μm CellTrics Filter (Partec, Münster, Germany). The filtered Boris-expressing cells were separated from non-transfected cells on the FACS Aria III device (BD Biosciences, Le Pont de Claix, France) using settings for maximum purity. Sorting was performed in staining buffer with an 85-μm nozzle, a 488-nm laser, a photomultiplier tube E, a 525-nm dichroic and a 543/22-nm excitation filter. Sorted cells were collected in DMEM containing 10% FCS. Usually, approximately 4.5% of transfected cells could be separated from non-transfected cells. Different approaches of transfected and non-transfected cells were performed: non-labeled, isotype control antibody and anti-Boris primary antibody.

### DNA methylation analysis by sodium bisulfite sequencing

For the sodium bisulfite conversion the EpiTect bisulfite kit (Qiagen) was used according to a modified protocol. One μg of genomic DNA supplemented with 35 μl DNA Protect buffer and 85 μl bisulfite mix were alternately denatured at 99°C and incubated at 60°C for 5, 25, 5, 85, 5 and 175 min. Following purification and concentration of the sodium bisulfite-treated DNA on an EpiTect column (Qiagen), 1 μl of the modified DNA was amplified with primers specific for MAGE-A1 and -A2 promoter fragments [6]. The PCR products were purified using the DNA Clean & Concentrator-5 kit (Zymo Research, Greiburg, Germany) and sequenced using the Big Dye Terminator v1.1 Cycle Sequencing kit (Applied Biosystems) on an automated Genetic Analyzer 3130 (Applied Biosystems).

### Chromatin immunoprecipitation (ChIP) assay

Exponentially growing MCF-7 cells stimulated by 5-aza-CdR (Sigma-Aldrich) and/or TSA (Sigma-Aldrich) as well as cells transfected by BORIS expression plasmid were used in ChIP experiments. The Magna ChIP™ G Chromatin Immunoprecipitation Kit (Millipore, Schwalbach, Germany) was carried out according to the manufacturer’s recommendations. Briefly, cells were fixed in 1% formaldehyde in minimal medium for 10 min at room temperature (RT) before being washed, scraped, and pelleted in ice-cold PBS. Cells were lysed with a hypotonic lysis buffer supplemented with a protease inhibitor cocktail for 15 min on ice, and nuclei were pelleted by centrifugation for 5 min, 2900 rpm at 4°C. The nuclei pellet was sheared in 500 μl nuclear lysis buffer supplemented with a protease inhibitor cocktail by sonication at 25% power for 4 min on ice (Sonicator UP50H; Dr. Hielscher GmbH, Teltow, Germany) to chromatin fragment lengths of 200 to 1000 bp. Aliquots of whole-cell lysates were saved as input DNA. The sonicated lysates were immunoprecipitated using 3 μg of either the control antibody IgG (Abcam, Cambridge, United Kingdom) or antibodies against acetylated histones H3 (H3K9ac) (Upstate) and H4 (H4K8ac) (Abcam), and methylated histones H3K4me, H3K4me2, H3K9me, H3K9me3, H4K20me, H4K20me2 and H4K20me3 (Upstate). Twenty μl magnetic beads (protein G, Millipore) were added to each reaction and incubated overnight at 4°C. After washing, the immunoprecipitants were recovered and incubated with proteinase K (Millipore) for 2 hours. The DNA fragments were purified on columns (Millipore) and eluted by 50 μl of elution buffer.

### Quantitative real-time PCR

Quantitative real-time PCR analysis was performed using the QuantiTect SYBR Green PCR kit system (Thermo Fisher, Schwerte, Germany) on a Realplex^4^ System Mastercycler Epgradient S (Eppendorf, Hamburg, Germany). Each reaction contained 2 μl cDNA or purified immunoprecipitated DNA fragments, 5 μl SYBR-Green PCR master mix and 4 pmol primer sets in a final volume of 10 μl. The DNA was amplified by the primer pairs specific for BORIS (5’-CTC AGG TGA GAA GCC TTA CG-3’ and 5’-TGA TGG TGG CAC AAT GGG-3’), MAGE-A1 (5’- GGC CGA AGG AAC CTG ACC -3’ and 5’-GTC CTC TGG GTT GGC CTGT-3’), β-Actin (5´-CCA ACC GCG AGA AGA TGA-3´ and 5´-CCA GAG GCG TAC AGG GAT AG-3´) and RPLP0 (housekeeping gene, ChIP, 5’-TTA GTT TGC TGA GCT CGC CAG-3’ and 5’-CTC TGA GCT GCT GCC ACC TG-3’). The following PCR cycling conditions were used: 95°C for 15 s, 58°C or 60°C for 30 s, and 72°C for 30 s, for 45 cycles. After amplification the specificity of PCR products was determined by melting curve analyses. For quantification a serial dilution of genomic DNA was generated and served as internal standard in each run. For the amplified immunoprecipitated DNA, the background of non-specific IgG immunoprecipitation was subtracted from the calculated ratio between the data derived from the histone-specific immunprecipitation and input DNA. Each sample was thermocycled in duplicate, and all experiments were repeated at least three times.

To analyze the expression patterns of BORIS isoforms in basal and BORIS-transfected MCF-7 and BCM1 cells, quantitative real-time PCR was performed as previously described [17]. BORIS isoforms were divided into six subfamilies, sf1 to sf6, based on their 39 sequences [17]. The Taqman probe sf1 was designed against sequences between exon 9 and 10 of the BORIS B0 and detects BORIS isoforms B0, B1, A1, A2, A3, and C1 (Additional file 1: Table S1). The absolute quantification approach was applied to estimate the actual number of BORIS transcripts detected by sf1 per 50 ng of total RNA. BORIS B1 contains a unique splice site that was used to design the sf5 probe, and the total number of B1 transcripts was subtracted from the total number of transcripts detected by the sf1 probe. The Taqman probe sf2 detects at least two BORIS isoforms, A4 and C2 that produce the same protein but are expressed from two alternative promoters, A and C, respectively. The Taqman sf3 probe detects five isoforms: A5, A6 B4, B5, and C6. The Taqman probe sf4 was designed to detect at least six BORIS isoforms: C3, B2, B3, C4, C5, and C8. The B1 isoform has a unique C-terminus and 3´UTR that were used to design the sf5 probe. The sf6 probe detects four BORIS isoforms: B6, B7, C7, and C9 [17].

### Expression of recombinant protein

For protein expression and purification the EnPresso™ Tablet Cultivation Set (BioSilta, Oulu, Finnland) and HaloTag® Protein Purification System (Promega) were used, respectively. To induce protein expression, a transformed culture of KRX competent cells (Promega) at an optical density of 9-13 at 600 nm was supplemented with a “booster solution” (EnZ I’m and 0.05% rhamnose). After centrifugation for 10 min at 5600 rpm and 4°C, the cell pellet was resuspended in HaloTag® Protein Purification buffer (50 mM HEPES, 150 mM NaCl, 1 mM DTT, 0.005% IGEPAL CA-630; Promega), 10 mg/ml lysozyme (Sigma-Aldrich) and RQ1 RNase free DNase (Promega) and disrupted by sonication at 60% power for 45 s on ice (Sonicator UP50H; Dr. Hielscher GmbH, Teltow, Germany). The proteins were purified from the sonicated cell lysates according to the manufacturer’s recommendations (Promega). Briefly, lysates were incubated with HaloLink™ resin, followed by washing with HaloTag® Protein Purification buffer and cleavage with TEV Protease Cleavage Solution (HaloTag® Protein Purification buffer supplemented with 1/16 volume TEV protease) on a rotator (NeoLab, Heidelberg, Germany) for 1 h at RT. After centrifugation 50 μl of 50% HisLink™ resin was added and incubated on the rotator for 20 min at RT. The supernatant contained the recombinant proteins.

### Pull down assay

Pull down assay was carried out according to the manufacturer’s recommendations for HaloLink™ resins (Promega). The “bait” HaloTag fusion proteins were prepared by incubating 1 μg FN19A (HaloTag®7) T7 SP6 Flexi vector (Promega) with *in vitro* TNT® Quick-coupled Transcription/Translation System (Promega) containing 40 μl TNT Quick Master mix and 1 mM methionine at 30°C for 90 min. The “prey” proteins were prepared by incubating 1 μg pcDNA3.1 constructs with TNT® Quick-coupled Transcription/Translation System (Promega) and 1000 Ci/mol labeled [^35^S]-L-methionine (Hartmann Analytic, Braunschweig, Germany) at 30°C for 90 min. For the assay 20 μl of each bait and prey proteins were mixed and incubated for 1 h at RT on a shaker. As a negative control 20 μl TNT Master mix were used instead of using the “bait” protein. The HaloLink™ resin was prepared by washing in binding buffer (100 mM Tris (pH 7.6), 150 mM NaCl and 0,05% IGPAL-630) three times. Twenty μl of bait-prey complex were added to the HaloLink^TM^ resin resuspended in 100 μl binding buffer. After incubation on a rotator for 90 min at 4°C, the complex was centrifuged and washed three times in wash buffer (100 mM Tris pH 7.6, 150 mM NaCl, 1 mg/ml BSA and 0.05% IGPAL-630). The bound proteins were separated on a 12% SDS polyacrylamide gel.

### Statistical analyses

The statistical analyses were performed using the SPSS software package, version 18.0 (SPSS Inc. Chicago, IL). Statistical difference of mRNA expressions was calculated using ANOVA with Dunnett test for all pairwise comparisons that correct for experiment-wise error rate. Missing data were handled by pairwise deletion. A p-value ≤0.05 was considered as statistically significant. All p-values are two-sided.

### Ethics statement

In the present manuscript, the research does not involve human subjects, human material, or human data, or used regulated vertebrates or invertebrates

## Results

### BORIS stimulates MAGE-A1 mRNA expression in MCF-7 and BCM1 cells

**Figure 1:**
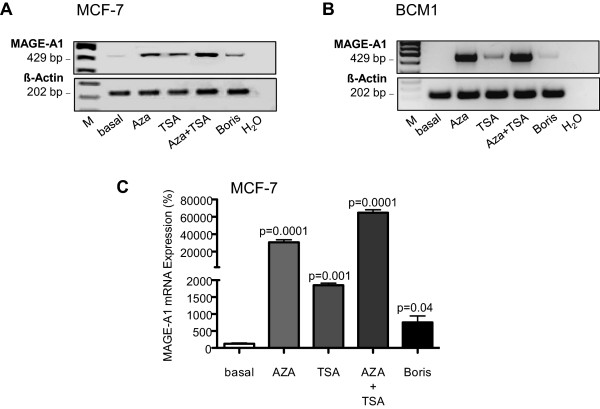
**Comparison of the MAGE**-**A1 mRNA expression in 5**-**aza**-**CdR**- **and**/**or TSA**-**stimulated MCF**-**7 and BCM1 cells with the expression in BORIS**-**transfected cells.** RT-PCR products of MAGE-A1 mRNA expression prior and after stimulation of MCF-7 **(A)** and BCM1 cells **(B)** with the demethylating agent 5-aza-CdR and/or the histone deacetylase inhibitor TSA or after transient transfection of these cells with an expression plasmid encoding for BORIS were separated on an agarose gel. The bar chart shows the relative changes in mRNA expression levels of MAGE-A1 in MCF-7 cells by quantitative real-time PCR. The significant p-values are shown **(C)**. H_2_O lane serves as a negative control. The housekeeping gene β-Actin was selected as an internal control due to the lack of influence of any stimulation involved.

We previously demonstrated that the demethylating agent 5-aza-CdR and the histone deacetylase inhibitor TSA synergistically upregulate MAGE-A1 expression in cell lines derived from different cancer types [6]. Moreover, Vatolin et al. reported that conditionally expressed BORIS induces expression of a series of CTA genes, including MAGE-A1 gene [14], but converse data have also been reported demonstrating that stable expression of BORIS in melanoma cell lines did not induce expression of MAGE-A1 [20]. In order to examine whether BORIS is actually able to activate the MAGE-A1 promoter and to which extent, we compared its influence with the stimulatory effect of 5-aza-CdR and/or TSA on MAGE-A1 transcription in cancer cell line settings. For our current investigations, we chose 3 breast cancer cell lines: MDA-MB-468, MCF-7 and BCM1 because of their different levels of MAGE-A1 and BORIS transcripts. As shown in Table 1 and measured by quantitative real-time PCR, MDA-MB-468 cells express relatively high levels of MAGE-A1 [2^(ΔCt) 19.33] and BORIS mRNA [2^(ΔCt) 48.78], whereas MCF-7 cells do not (or negligibly) express MAGE-A1 mRNA [2^(ΔCt) 2.00] and express low levels of BORIS [2^(ΔCt) 6.92 with a high standard deviation]. In the micrometastatic cell line BCM1, the expression of both genes is opposite: no levels of MAGE-A1 [2^(ΔCt) 1.07] and high levels of BORIS [2^(ΔCt) 24.39]. We transiently transfected expression plasmid encoding BORIS into both cell lines, with negligible transcript levels of MAGE-A1, and quantified endogenous MAGE-A1 mRNA by RT (reverse transcription)-PCR and gel electrophoresis. As depicted in Figure 1, BORIS was able to stimulate or induce the expression of MAGE-A1 in MCF-7 cells (Figure 1A) and BCM1 (Figure 1B) cells. In both cell lines, the BORIS-mediated stimulation was much weaker than the stimulatory effect by both agents (5-aza-CdR and/or TSA, Figure 1). Performing real-time PCR, we found that 5-aza-CdR (p = 0.0001), TSA (p = 0.001), 5-aza-CdR plus TSA (p = 0.0001) and BORIS (p = 0.04) stimulated the RNA expression 30-, 18-, 60- and 7-fold, respectively, in MCF-7 cells (Figure 1C). This ostensibly weaker activation by transfected BORIS may be partly due to the fact that transfection efficiency is usually much lower and about 10% (as deduced from FACS analyses and shown later), but 5-aza-CdR and TSA treatment can affect 100% of cells taken into experiment.

**Table 1:** **Relative expression levels of MAGE**-**A1 and BORIS mRNA in breast cancer cell lines as measured by quantitative real**-**time PCR**

Cell lines	MAGE-A1	BORIS
MDA-MB-468	19.33 ± 3,84 (high)	48.78 ± 3.38 (high)
MCF-7	2.00 ± 0.87 (no)	6.92 ± 3.86 (low)
BCM1	1.07 ± 0.35 (no)	24.39 ± 3.34 (high)

The relative mRNA expression levels were evaluated by the ΔCt method as follows: ΔCt = Ct value of reference RPLPO - Ct value of mRNA of interest. The relative expression levels of the mRNA of interest corresponded to the 2^(ΔCt)*1000 value.

### Knock-down of BORIS mRNA reduces the transcript levels of MAGE-A1

**Figure 2:**
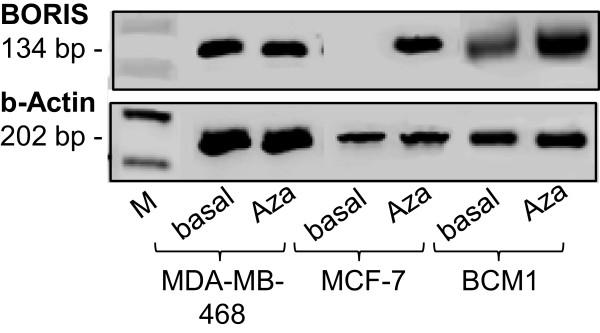
**BORIS mRNA expression in MDA**-**MB**-**468**, **MCF**-**7 and BCM1 cells,** **untreated or treated with 5**-**aza**-**CdR.** RT-PCR products of BORIS mRNA were separated on an agarose gel.

To further evaluate the stimulatory effect of BORIS on MAGE-A1 gene expression, we carried out knock-down experiments in MDA-MB-468 and MCF-7 cells. First, we determined the expression levels of BORIS in MDA-MB-468, MCF-7 and BCM1 cells by RT-PCR and gel electrophoresis. As expected, we found a similar expression profile of BORIS mRNA (Figure 2) to that detected by quantitative real-time PCR (Table 1). However, gel electrophoresis and quantitative real time showed no and low expression levels of BORIS in MCF-7 cells, respectively, but the tendency was similar. The additional stimulation with 5-aza-CdR showed that induction of BORIS expression may occur by DNA demethylation (Figure 2).

**Figure 3:**
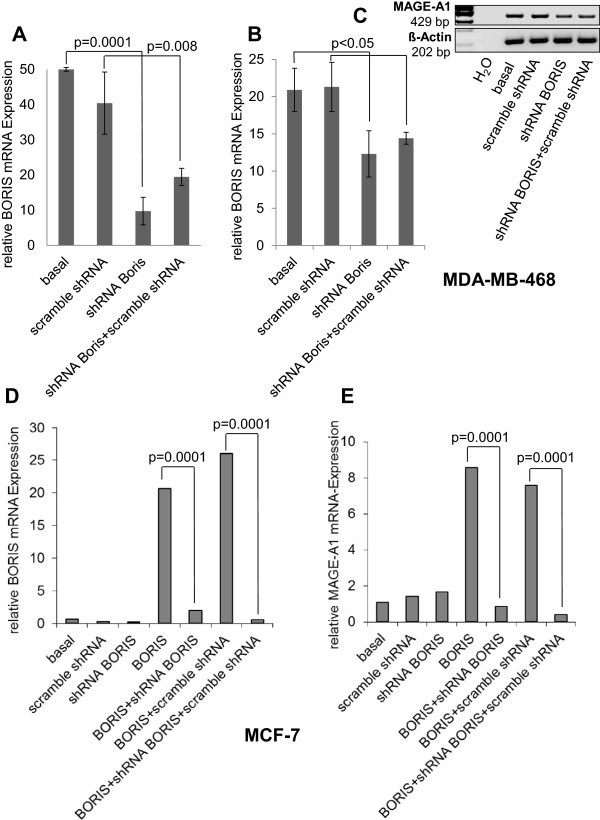
**BORIS**-**specific shRNA knocks down BORIS and decreases MAGE**-**A1 gene expression.** MDA-MB-468 **(A, B, C)** and MCF-7 **(D, E)** cells were transiently transfected with expression plasmid containing BORIS-specific shRNA and control plasmid encoding for a scramble shRNA. In contrast to MDA-MB-468 cells with their high levels of endogenous MAGE-A1 and BORIS mRNA levels, MCF-7 cells showing no expression of MAGE-A1 were additionally cotransfected with the expression plasmid containing the BORIS sequence. After a 48 hour transfection, mRNA levels were measured by PCR. Changes in mRNA expression levels of BORIS **(A)** and MAGE-A1 **(B)** by quantitative real-time PCR and MAGE-A1 by gel electrophoresis **(C)** in MDA-MB-468 cells. Real-time PCR derived changes in mRNA expression levels of BORIS **(D)** and MAGE-A1 **(E)** in MCF-7 cells. The significant p-values are shown.

We knocked down the high expression of endogenous BORIS in MDA-MB-468 cells by a BORIS specific shRNA cassette. The transfection with a plasmid encoding for a scramble shRNA served as a control. At 48 or 72 hour post-transfection, we quantified the changes in the BORIS and MAGE-A1 mRNA levels by quantitative real-time PCR and RT-PCR/gel electrophoresis. As measured by real-time PCR, BORIS-specific shRNA reduced the endogenous BORIS mRNA expression from 100% down to 20% in basal MDA-MB-468 cells (p = 0.0001) and, documenting more the specificity of the experiment, from 75% down to 40% in MDA-MB-468 cells transfected with the control plasmid encoding for scramble shRNA (Figure 3A, p = 0.008). As shown by quantitative real-time PCR (Figure 3B, p < 0.05) and on an agarose gel (Figure 3C), the BORIS-specific shRNA (with and without scramble shRNA) downregulated the basal endogenous MAGE-A1 expression approximately 30%. We also carried out these knock-down experiments in MCF-7 cells that were additionally transfected with an expression plasmid encoding for BORIS. Therefore, we co-transfected MCF-7 cells with an expression plasmid encoding for BORIS, to upregulate MAGE-A1 expression in this cell line. BORIS-specific shRNA reduced the BORIS mRNA expression nearly completely in presence and absence of scramble shRNA (Figure 3D, p = 0.0001). Likewise, the downregulation of MAGE-A1 expression by BORIS-specific shRNA was more prominent in MCF-7 cells than in MDA-MB-468 cells. As measured by quantitative real time PCR, BORIS-specific shRNA reduced the MAGE-A1 expression down to 10% in presence and absence of scramble shRNA (Figure 3E, p = 0.0001). This stronger downregulation of BORIS and MAGE-A1 in MCF-7 cells is caused by the overexpression of BORIS in these cells, whereas the analyses in MDA-MB-468 were carried with endogenous BORIS.

These results show that changes in the BORIS transcript levels are associated with those of MAGE-A1 and corroborate that BORIS is involved in the activation of MAGE-A1 gene expression.

### BORIS affects the DNA methylation pattern of MAGE-A1 gene

Promoter hypermethylation is responsible for the restricted expression of the tumor-associated MAGE-A antigens. It was reported that DNA demethylation of the Ets-1 binding sites of the MAGE-A1 promoter is sufficient to activate gene expression [5]. In addition, the transcriptional start site located in the region between -30 and +30, and responsible for basal activity of the MAGE-A1 promoter, should be demethylated for the induction of MAGE-A expression [25]. Previously, we investigated the influence of the DNA demethylation agent 5-aza-CdR together with the histone deacetylase inhibitor TSA on the mRNA expression of MAGE-A1 gene and the other family members (MAGE-A2, -A3 and -A12) in different cell lines. Moreover, we assessed the methylation status of the MAGE-A promoters by sodium bisulfite mapping before and after stimulation with the demethylating agent 5-aza-CdR and/or TSA. While the methylation patterns clearly correlated with the basal MAGE RNA transcript levels, up-regulation of MAGE-A expression mediated by 5-aza-CdR resulted in a reduction in promoter methylation ranging between 1% and 19%. Although TSA was able to synergistically enhance 5-aza-CdR-mediated MAGE-A transcription, we could not observe further DNA demethylation with both substances (5-aza-CdR + TSA) together [6]. This heterogeneous DNA methylation pattern could be caused by the heterogeneous and random spreading of the demethylating agent in the cells, and the insensitivity of some cells to these agents.

In respect to the inducing effect of BORIS on MAGE-A1 mRNA expression, it was of interest to examine the influence of BORIS on methylation pattern of the MAGE-A1 promoter. In the present study, we compared the DNA methylation patterns of the promoter in MCF-7 cells transfected with the expression plasmid encoding for BORIS to the pattern in non-transfected and untreated cells. For these experiments, we chose, therefore, MCF-7 cells, because they do not express MAGE-A1 mRNA (Table 1). Based on the usually low transfection efficiency we sorted the transfected MCF-7 cells from untransfected cells by FACS and observed a transfection efficiency of about 10%. Subsequently, sodium bisulfite mapping showed a demethylation of the MAGE-A1 promoter of approximately 56% (range from 44 to 69%) in the sorted BORIS-transfected cells, compared with the sorted non-transfected and untreated MCF-7 cells. As shown by two examples of the supplementary Additional file 2: Figure S1, BORIS demethylated the binding sites for Ets-1, Sp1 and BORIS which are essential for the activation of MAGE-A1.

### Histone modifications at the promoter of MAGE-A1

Besides DNA methylation, histone modifications also have an impact on promoter activity. In general, acetylation of N-terminal histone tails is a dominant signal for active chromatin facilitating the binding of components of the basal transcription machinery and transcription factors [26]. Histone methylation can be either an active or repressive signal. Mono-, di- and trimethylation of H3K4 are involved in gene expression [9]. The monomethylations of H3K9 and H4K20 are linked to gene activation, whereas trimethylations of these histones at lysine residues are linked to repression [27].

**Figure 4:**
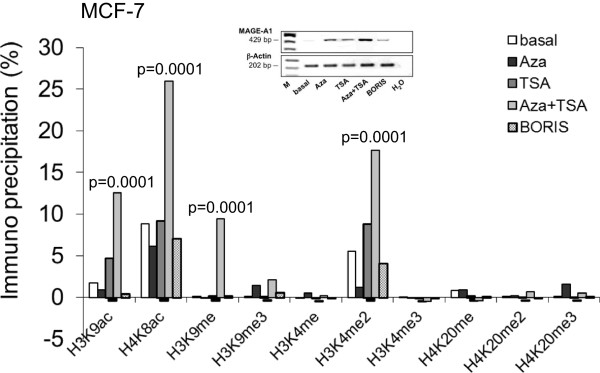
**Histone signature at the MAGE**-**A1 promoter as examined by chromatin immunoprecipitation.** DNA was derived from unstimulated (basal) MCF-7 cells, 5-aza-CdR- and/or TSA-stimulated MCF-7 cells and MCF-7 cells transfected with the expression plasmid encoding for BORIS. DNA-bound histones were immunoprecipitated by antibodies specific for methylated and acetylated histones, and amplified in a real-time PCR by a primer pair specific for the MAGE-A1 promoter. The background of the non-specific IgG immunoprecipitation was subtracted from the calculated ratio between the data derived from the histone-specific immunoprecipitation and input DNA. H3K9, Lysine 9 of histone H3; H4K8, Lysine 8 of histone H4; H3K4, Lysine 4 of histone H3; H4K20, Lysine 20 of histone H4; ac, acetylated; me, monomethylated; me2, dimethylated; me3, trimethylated. The significant p-values are shown.

Since BORIS may demethylate the MAGE-A1 promoter, we also analyzed its impact on the modifications of histones bound at the MAGE-A1 promoters. To investigate the changes in the histone signature of MAGE-A1 promoter, it was compared in basal MCF-7 cells (no expression of MAGE-A1, Table 1) to the signature in MCF-7 cells stimulated by 5-aza-CdR with/without TSA or transfected with the expression plasmid encoding for BORIS. For these analyses we used antibodies specific for acetylated histones H3K9 and H4K8, and for methylated histones H3K4, H3K9 and H4K20. We performed immunoblot analyses and documented specific recognition of histone modifications by these specific antibodies. The histone modifications could not be determined in the micrometastatic BCM1 cells because of their slow cell growth and high cell death caused by 5-aza-CdR and TSA. Upon treatment of MCF-7 cells with TSA, an enrichment of H3K9ac could be observed, indicating the function of TSA as histone deacetylase inhibitor (p = 0.001). While DNA demethylation by 5-aza-CdR had no or a minor effect on the histone modifications, 5-aza-CdR and TSA were able to enrich H3K9ac, H4K8ac, H3K9me, and H3K4me2 (p = 0.0001). Based on their low levels, the relative changes in the histone modifications of H3K4me3, H4K20me, H4K20me2 and H4K20me3 could not be evaluated, but did not seem to be significant (Figure 4). Due to the nature of the experimental procedures of ChIP, we could not sort transfected cells from untransfected cells by FACS analyses. Therefore, the predominant occurrence of untransfected cells in the transfection assay (10% of transfection efficiency) may be the reason, that we could not observe any alterations in the histone modifications mediated by transfected BORIS (Figure 4).

### Differential effects of transcription factors BORIS, Sp1 and Ets-1 in the regulation of MAGE-A1 expression

**Figure 5:**
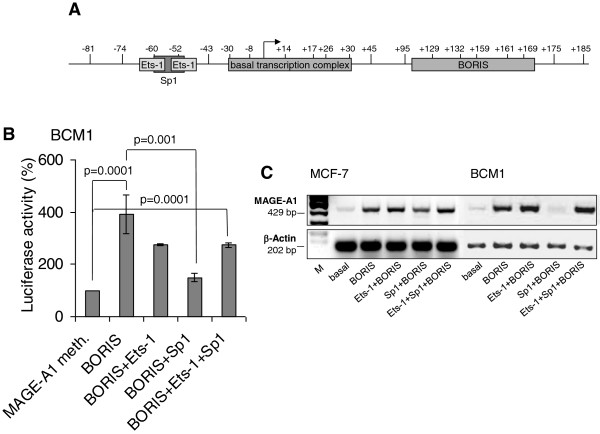
**MAGE**-**A1 promoter activity in basal and transfected cancer cells.** Schematic view of the MAGE-A1 promoter fragment (-81/-185). The binding sites for Ets-1, Sp1 and basal transcription complex are indicated by grey boxes. The start site is indicated by an arrow. The vertical lines with the numbers mark the cytosine in the CpG dinucleotides **(A)**. Luciferase activity of the *Hpa*II-methylated plasmid containing the MAGE-A1 promoter fragment (-77/+183) in BCM1 cells which were transiently co-transfected with expression plasmids encoding for BORIS, Ets-1 and Sp1. The basal MAGE-A1 promoter activity was set to 100%. The activities derived from the reference plasmid encoding for the Renilla Luciferase were used to normalize the variability in transfection efficiency. The significant p-values are shown **(B)**. Endogenous mRNA expression of MAGE-A1 in MCF-7 and BCM1 cells basal or transfected with expression plasmids encoding for BORIS, Ets-1 and Sp1 as determined by RT-PCR and gel electrophoresis. The housekeeping gene β-Actin was selected as an internal control due to the lack of influence of any stimulation involved **(C)**.

In order to functionally investigate the impact of BORIS on promoter settings, we examined the influence of BORIS on the activity of the methylated MAGE-A1 promoter in the context of transcription factors Ets-1 and Sp1. We transiently co-transfected methylated reporter plasmids pGL2/MAGE-A1 (-77/+183) containing the BORIS binding site located downstream of the start site (Figure 5A), and expression plasmids encoding for BORIS, Ets-1 or Sp1 into BCM1 cells. As expected, aberrantly expressed transcription factors Ets-1 and Sp1 had no effect on methylated MAGE-A1 [11]. However, transfected BORIS was able to activate the methylated promoter in these cells (p = 0.0001). Overexpression of Ets-1 could not further upregulate the promoter activity mediated by BORIS, as shown by several repetitions. Surprisingly, co-transfection with an expression plasmid encoding for Sp1 partly repressed the stimulatory effect mediated by BORIS (p = 0.001), whereas the addition of expression plasmid encoding for Ets-1 abrogated this repression (Figure 5B).

To verify the repressive effect of Sp1 on the BORIS-activated MAGE-A1 promoter, we transiently transfected the expression plasmids into MCF-7 and BCM1 cells, and analyzed the endogenous MAGE-A1 mRNA level by gel electrophoresis. Ectopic expression of BORIS could induce the mRNA expression of MAGE-A1 in both cell lines (Figure 5C). This upregulation could be slightly increased by the co-expression of Ets-1. In contrast, exogenous Sp1 reversed the stimulatory effect mediated by BORIS on MAGE-A1 mRNA transcription. We repeated the experiments on transcription of MAGE-A1 several times with similar results (Figure 5C). These findings support our data obtained from the reporter luciferase activity measurements (Figure 5B) and indicate a differential regulation of the MAGE-A1 by BORIS and Sp1.

To compare the transfection efficiency and assess if similar amounts of the different expression plasmids entered BMC1 and MCF-7 cells, we performed real-time PCR using primers that specifically bind to the expression plasmids and the inserted genes (BORIS, Ets-1 and Sp1). We found a CT value of 27.3 (STD 0.3) for BORIS, 27.3 (STD 0.2) for Ets-1, 27.8 (STD 0.2) for Sp1, indicating a similar inclusion of the expression plasmids.

### Ectopic BORIS expression upregulates its alternatively spliced transcripts from its own genomic locus

**Figure 6:**
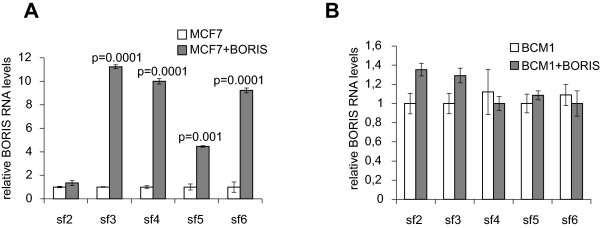
**Upregulation of alternatively spliced BORIS transcripts in MCF-** **7 and BCM1 cells as determined by quantitative real time PCR.** Changes in mRNA expression levels of BORIS isoforms sf2 to sf6 in MCF-7 **(A)** and micrometastatic breast cancer BCM1 cells **(B)**, which were basal or transiently transfected with the expression plasmid containing the BORIS (sf1) sequence. The significant p-values are shown.

The differential regulation of MAGE-A1 gene expression could be explained by a separate site in MAGE-A1 promoter that could be recognized by a BORIS alternatively spliced isoform with different sequence specificity. For this reason, we compared mRNA patterns of alternatively spliced BORIS isoforms in basal MCF-7 and BCM1 cells with analogous cells transfected with BORIS (the first cloned, original form B0). As we recently described [17], BORIS isoforms could not be specifically discriminated by quantitative RT-PCR because most isoforms share sequence similarities, making it impossible to design primers and probes that would detect each BORIS isoform as a separate species. Therefore, we operationally divided the 23 isoforms into six subfamilies (sf1 to sf6) based on their unique 3’ terminal sequences, which were used to design 6 Taqman probes for quantitative real-time PCR [17]. With the exception of sf1, which detects the original BORIS form (B0) that was transfected into the cell lines, we measured the relative units of BORIS isoforms sf2 to sf6 in basal and BORIS-transfected MCF-7 and BCM1 cells. As shown in Figure 6, BORIS stimulated substantially its isoforms sf3 to sf6 in MCF-7 cells which do not express endogenous BORIS (Figure 6A, p = 0.0001), whereas BORIS only stimulated weakly sf2 and sf3 in BCM1 cells which express high levels of endogenous BORIS (Figure 6B).

### Interaction of transcription factors BORIS and Sp1 with the TATA binding protein

**Figure 7:**
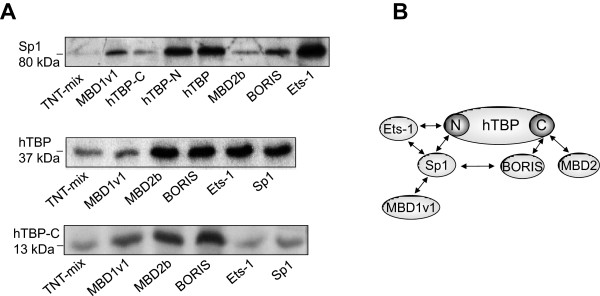
**BORIS and Sp1 interact with the TATA binding protein** **(hTBP).** The methionine labeled “prey” fusion proteins Sp1, hTBP and the carboxyl terminus of hTBP (hTBP-C) were incubated with various resin-bound “bait” proteins as indicated below the polyacrylamide gels. The protein complexes were collected by precipitation and separated by gel electrophoresis. The weak band in the TNT-mix (negative control) is unspecific and serves as a background level **(A)**. Schematic view of the detected protein-protein interactions. N, amino terminus; C, carboxyl terminus **(B)**.

In our recent publication, we have shown the cooperative and competitive interplay of transcription factors (Ets-1, Sp1) and epigenetic factors (BORIS, MBD1, MBD2a) to activate or repress the MAGE-A1 promoter by transient transfection assays [11]. To determine if these proteins, together with BORIS, can form secondary complexes with each other in the absence of DNA and to understand better the mechanism underlying the regulation of MAGE-A1 expression, we carried out *in vitro* protein-protein interaction assays. Each of the proteins was either a resin-bound “bait” fusion protein or a [^35^S]-L-methionine labeled “prey” protein. As shown by the separation of the bound proteins on polyacrylamide gels and previously reported [28, 29], the transcription factor Sp1 efficiently interacted with Ets-1 and the amino-terminus of the human TATA binding protein (hTBP), the general transcription factor of the basal transcription complex. We show for the first time, that Sp1 also interacted with BORIS and MBD1v1, which up- and downregulate MAGE-A1 promoter activity, respectively, but this protein-protein interaction was much weaker than its interaction with Ets-1 and hTBP amino-terminus. Conversely, hTBP interacted with MBD2b, BORIS, Ets-1 and Sp1 (Figure 7A). For the interaction with MBD2b and BORIS the evolutionarily conserved carboxyl (C) terminus of hTBP was necessary and sufficient. At present, it is unclear whether these interactions with hTBP play a functional role in the competitive transcriptional regulation of Sp1 and BORIS. However, this observation is supported by our ChIP-seq data on several cancer cell lines – BORIS sites are frequently overlapped with hTBP [17]. For a better overview, Figure 7B schematically summarizes the protein-protein interactions.

## Discussion

In the current study devoted mainly to understanding the transcriptional regulation of MAGE-A1, we detected that ectopically expressed BORIS was able to induce MAGE-A1 promoter activity in MCF-7 cells and micrometastatic BCM1 cells. This activation by BORIS was associated with DNA demethylation of the MAGE-A1 promoter. It is of interest that MAGE-A1 activation by BORIS may vary depending on cell line but the strongest upregulation occurs when there is already low expression level of MAGE-A1. If the expression is completely shut down as in normal primary cells, activation is also observed but at a very low level and, likely, in a fraction of cells. These data suggest that the epigenetic landscape of cells is different and their responsiveness to ectopic transcription factors is also different. In our present study, we describe the complex interplay of general transcription and epigenetic factors in MAGE-A1 regulation. Exogenous Sp1 partly repressed the induction of MAGE-A1 gene expression mediated by BORIS. Ets-1 could not further upregulate the BORIS-mediated promoter activity, but Ets-1 abrogated the Sp1-mediated repression in reporter assays. As the two Ets-1 sites overlap the Sp1 site, it appears to be rather simple to postulate 1) that the opposite effects of those two factors are due to their interference on the promoter, and 2) Ets-1 effects are stronger probably because there are two Ets-1 sites compared to one Sp1 site in the MAGE-A1 promoter. The BORIS site is located about 150 bp downstream to the Sp1 site, in the first exon of MAGE-A1. As Sp1 and BORIS have independent target sites around the start site of the MAGE-A1 promoter, the opposite effects of those two factors need to be mechanistically explained. Thus, the competitive interplay between BORIS and Sp1 does not seem to be caused by alternation of the DNA binding of both factors. However, the physical protein-protein interaction between BORIS and SP1, which we observed and was also recently reported [30], might diminish the BORIS-mediated activation of MAGE-A1.

The repressive effect by Sp1 could also be favored by its additional interaction with the epigenetic factor MBD1v1. It is still unclear whether MBD1v1 exerts its repressive effect by interaction with Sp1 at the Sp1 binding site of the promoter or by preventing the binding of Sp1 to its motif. Our preliminary data suggest that the third cysteine-rich CXXC domain of MBD1v1 is involved in this interaction, because the splice variant MBD1v3, which lacks this domain, did not bind to Sp1. The ability of MBD1v1 to repress unmethylated promoters also depends on this third CXXC domain [12]. In this regard, our recent binding analyses of EMSA showed that MBD1v1 does not only bind to methylated, but also to unmethylated MAGE-A promoters and is able to repress unmethylated MAGE-A promoters [11]. This *in vitro* ability is also supported by a previously published study demonstrating a methylation-independent repression by MBD1v1 on another promoter [31]. Our data are of note, because co-transfection of Ets-1 or Sp1 did not lead to an abrogation of MBD1v1-mediated repression [11]. In respect to the potential role of MBD1v1 as a repressor of unmethylated MAGE-A promoters and to the mechanisms underlying the transcriptional repression, we investigated whether MBD1v1 can also interact with Ets-1. Due to problems in the production of recombinant Ets-1 protein, we could not determine if Ets-1 can form a secondary complex with MBD1v1.

How does the binding of Sp1 partly abrogate BORIS activation? Currently, we can suggest either direct interaction of BORIS with Sp1 generating a DNA kink that diminishes transcription, or a separate site in MAGE-A1 promoter that could be recognized by a BORIS alternatively spliced isoform with different sequence specificity. That site might overlap with the Sp1 site and thus directly interfere with the Sp1 transcription factor. Ets-1 could stabilize BORIS isoform binding preventing Sp1 to act at this site. We show here that BORIS can activate transcription of its own locus. However, the strength of this activation depends on the cell line and likely on other transcription factors present in a particular cell type. More research is required to clarify this phenomenon.

Both BORIS and Sp1 are capable of binding to different domains of hTBP, the general transcription factor of the basal transcription machinery, which forms a preinitiation complex with RNA polymerase II to start mRNA transcription. Our data are in line with previously published data showing that Sp1 interacts with the N-terminus of hTBP [32]. In contrast, the interaction between BORIS and hTBP takes place with the C-terminal evolutionarily conserved domain of hTBP, which has been shown to interact with several other transcription factors [32, 33]. One could envisage that the MAGE-A1 promoter activity mediated by BORIS might be based on its interaction with the general transcription factor hTBP. However, to support this hypothesis, further experiments, such as luciferase assays, are to be assessed.

Furthermore, we demonstrate for the first time that the epigenetic factor MBD2 also interacts with the C-terminal domain of hTBP. In our recent study, we reported that although MBD2a had no binding activity to MAGE-A1 promoters in EMSA and ChIP assays, transfected MBD2a could stimulate luciferase activity of the unmethylated reporter plasmids containing the MAGE-A promoter fragments in a variety of cancer cell lines [11]. Fujita et al. explained the lack of binding activity of MBD2a and its activating effect for cAMP-responsive genes by the interaction of MBD2a with the RNA helicase A. They depicted a hypothetical model illustrating MBD2a as a factor in the transcriptional co-activator complex which is associated with RNA polymerase II [13]. We complement this model and show that MBD2 may interact with hTBP. This link with the basal transcription machinery could explain how MBD2a promotes MAGE-A transcription.

Functional studies reported that BORIS is neither necessary nor sufficient for DNA hypomethylation and activation of CTA genes in melanoma and ovarian cell lines, and additional factors are likely required for CTA antigen expression [20, 34]. Those data are in contrast to our previous data, which showed that aberrantly expressed BORIS is responsible for DNA demethylation and subsequent activation of most CTAs, including MAGE-A1, in carcinomas [14]. Our present observations support the hypothesis that BORIS may demethylate the MAGE-A1 promoter, upregulate the promoter activity and induce mRNA expression. In particular, BORIS seems to participate in DNA demethylation of the binding sites for the transcription factor Ets-1 and the transcriptional start site for the basal transcription complex. These target sites essential for the activation of MAGE-A1 are methylated in MAGE-A-negative cells.

Covalent modifications associated with histone tails are also involved in regulation of gene expression. The histone code dictates the recruitment of specific factors that in turn define the formation of open or closed chromatin structures [35, 36]. We could endorse the function of TSA as histone deacetylase inhibitor, because TSA was able to enrich acetylated H3K9. Our investigations also showed that both agents, 5-aza-CdR and TSA, were necessary to launch distinct histone modifications at the MAGE-A1 promoter. However, we found no effect of BORIS on the histone code. This could be explained by the low transfection efficiency of 10%. Recently, Bhan et al. showed that BORIS is able to change the histone code at the promoters of the MAGE-A members MAGE-A2, -A3 and -A4. In bronchial cancer cells, BORIS induction resulted in increased amounts of BORIS and activating histone modifications at these promoters along with a corresponding increase in CTA expression. Whereas BORIS binding at these promoters correlated with enrichment of activating modifications, absence of BORIS was associated with enrichment of repressive histone codes [37]. Moreover, it was reported that BORIS helps to recruit histone (H3K4) methyltransferase, SET1A, onto the promoters of myc and BRCA1 to promote a permissive histone modification status [21].

## Conclusions

In conclusion our data show that BORIS may demethylate and activate the MAGE-A1 promoter. The induction of the MAGE-A1 mRNA expression could be provoked by the interaction between BORIS and hTBP. Sp1 could partly repress the BORIS-mediated stimulatory effect by its direct interaction with BORIS and MBD1v1. Moreover, the secondary complex formation of MBD2 with hTBP may also play a role in the activation of MAGE-A transcription. However, more experiments have to be done to clarify the interplay of these factors in the regulation of MAGE-A genes expression.

## Electronic supplementary material

Additional file 1: Table S1: The primers and the Taqman probes used in quantitative real-time RT-PCR. (DOC 32 KB)

Additional file 2: Figure S1: Bisulfite-treated MAGE-A1 promoter sequence in sorted non-transfected and sorted BORIS-transfected MCF-7 cells. (DOCX 46 KB)

## Abbreviations

BORIS: Brother of the Regulator of Imprinted Sites
ChIP: Chromatin immunoprecipitation assay
CTA: Cancer-testis antigens
fetal calf serum: FCS
MAGE-A: Melanoma-associated antigen A
MBDs: Methyl-CpG binding proteins
hTBP: Human TATA binding protein
RT: Reverse transcription
TSA: Trichostatin A
5-aza-CdR: aza-2´-deoxycitidine.
