# Supplementary material for: Differential regulation of MAGE-A1 promoter activity by BORIS and Sp1, both interacting with the TATA binding protein
Source: BMC Cancer. 2014 Nov 3;14:796. doi: 10.1186/1471-2407-14-796 (PMC4230356; doi:10.1186/1471-2407-14-796)
Supplement: Supplementary file 1 — Additional file 1: Table S1: The primers and the Taqman probes used in quantitative real-time RT-PCR. (DOC 32 KB) [file 12885_2014_4979_MOESM1_ESM.doc]

**Additional file 1: Table S1.** The primers and the Taqman probes used in quantitative real-time RT-PCR.

| Probe | Transcript | Primer sequence  Forward(For):5’-3’; Reverse (Rev):5’-3’ | Tagman probe sequence  *6FAM- MGBNFQ* |
| --- | --- | --- | --- |
| **Probe BORIS (sf1)** | ***BORIS, BORIS A1, A2, A3, C1*** | **For: GCCACAAAGGGTCAGAAGGA**  **Rev: TCCTCAGCAGCAGCTTCGT** | **TGCGAAGGGATGGAA** |
| **Probe BORIS (sf2)** | ***BORIS A4, C2*** | **For: CAAGTATGCCAGTGTGGAGGTAAA**  **Rev: CAGCCTCTACTAAGATGCCATGAA** | **CCATTCTTGGACTTGAA*G*** |
| **Probe BORIS (sf3)** | ***BORIS A5, A6, B4, B5, C6*** | **For: AGTGCAAGTAGGAAGCCATGGT**  **Rev: GGCTGTTGTGCCGCTTTT** | **CCTTTTGCAGTCTAGTCC** |
| **Probe BORIS (sf4)** | ***BORIS C3, B2, B3, C4, C5, C8*** | **For: CCTGTGCCTGGCCTGATG**  **Rev: GGGAGACAAGAAGACCCAGTTC** | **GTTTTGTGGATCTCAGTGTT** |
| **Probe BORIS (sf5)** | ***BORIS B1*** | **For: GAAGGCGTGACCTGTGAAATG**  **Rev: TCCTGTACAGCCTGCGGAAT** | **TCCTCAACACGATGGAT** |
| **Probe BORIS (sf6)** | ***BORIS B6, B7, C7, C9*** | **For: TTTATTTAGCAGTAAGAGAGTCTGCATAGAT**  **Rev: CTACGGAAGCAAATACTTTGTGTTTT** | **CTGTGCCACAACCCCACTGTGTGG** |
