# Supplementary material for: Differential regulation of MAGE-A1 promoter activity by BORIS and Sp1, both interacting with the TATA binding protein
Source: BMC Cancer. 2014 Nov 3;14:796. doi: 10.1186/1471-2407-14-796 (PMC4230356; doi:10.1186/1471-2407-14-796)
Supplement: Supplementary file 2 — Additional file 2: Figure S1: Bisulfite-treated MAGE-A1 promoter sequence in sorted non-transfected and sorted BORIS-transfected MCF-7 cells. (DOCX 46 KB) [file 12885_2014_4979_MOESM2_ESM.docx]

**Additional file 2: Figure S1**

**Bisulfite-treated MAGE-A1 promoter sequence in**

**Sorted non-transfected and untreated MCF-7 cells** (16 CG dinucleotides: 16 methylated):

Ets-1 Sp1 Ets-1

TTTTTATTTAGGTNNGATTTGGTTTTCGTTAGGAAATANTCGGGTGTTCGGATGTGACGTTATTGATTTGCGTATTGTGGGGTANAGAGAAGCGAGGTTTTTNTTTTGAGGGACGGCGTAGAGTTCGGTCGAANGANTTTGATTTNGGTTTTGCGAGGAGGTAAGGTGAGAGGTTGAGGGANGNNNANNNNNCGTTNTTTTAAATAGAGAGTTTTAAATATTTTAGCGTCGTTTTTGNTGNTAGTTTTGGTTTATTCGCGGGAAGACGTTTTAGTTTGGGTTGNTTTTAGATTTTTGTTTTAAAAGTTTTGAGAGANATTAGGGGTA

Ets-1 Sp1 Ets-1

TTTTTATTTAGGTAGGATTTGGTTTTCGTTAGGAAATANTCGGGTGTTCGGATGTGACGTTATTGATTTGCGTATTGTGGGGTANANAGAAGCGAGGTTTTTATTTTGANGGACGGCGTAGAGTTCGGTCGAANGAATTTGATTTNGGTTTTGTGAGGGGGTANGGTGNNNGGTTGANGNNNNNNNGNTTCGTTATTTTAAATAGAGAGTTTTAAANATTTTAGCGTCGTTTTTGTTGNTAGTTTTGGTTTATTCGCGGGAAGACGTTTTAGTTTGGGTTGTTTTTAGNNTTTTGNTTTAAAAGTTTTGAGAGNNATTAGGGGTA

**Sorted BORIS-transfected cells** (16 CG dinucleotides: 5-6 methylated):

Ets-1 Sp1 Ets-1

TTTTTATTTAGGTAGGATTTGGTTTTTGTTAGGAAATATTTGGGTGTTTGGATGTGATGTTATTGATTTGCGTATTGTGGGGTAGAGAGAAGTGAGGTTTTTATTTTGAGGGACGGCGTAGAGTTCGGTTGAAGGAATTTGATTTAGGTTTTGTGAGGAGGTAAGGTGAGAGGTTGAGGGAGGATTGAGGATTTCGTTATTTTAAATAGAGAGTTTTAAATATTTTAGTGTTGTTTTTGTTGTTAGTTTTGGTTTATTTGTGGGAAGATGTTTTAGTTTGGGTTGTTTTTAGATTTTTGTTTTAAAAGTTT

Ets-1 Sp1 Ets-1

TTTTTATTTAGGTAGGATTTGGTTTTTGTTAGGAAATATTTGGGTGTTTGGATGTGATGTTATTGATTTGCGTATTGTGGGGTAGAGAGAAGCGAGGTTTTTATTTTGAGGGACGGCGTAGAGTTCGGTCGAAGGAATTTGATTTAGGTTTTGTGAGGAGGTAAGGTGAGAGGTTGAGGGAGGATTGAGGATTTTGTCATTTTAAATAGAGAGTTTTAAATATTTTAGTGTTGTTTTTGTTGTTAGTTTTGGTTTATTTGTGGGAAGNTGTTTTAGTTTGGGTTGTCTTTAGATTTTTGTTTTAAAAGTT

The arrow indicates the start site. The BORIS binding site is underlined. Methylated CG dinucleotides in dark grey and demethylated CpG dinucleotides (TpG) in light grey.
